# Supplementary material for: Pitfalls and windfalls of detecting demographic declines using population genetics in long‐lived species
Source: Evol Appl. 2024 Jul 14;17(7):e13754. doi: 10.1111/eva.13754 (PMC11246600; doi:10.1111/eva.13754)
Supplement: Supplementary file 1 — Appendix S1 [file EVA-17-e13754-s001.docx]

Supplemental Figure 1: Plots show percent detection in $\theta_{W}$ and $\pi$ between oldest and youngest individuals for perennial models (*A* = 2, 5, 10, 20). The old age bin is made up of individuals whose age at a given timepoint fell above the 50^th^ quantile. The young age bin is made up of the youngest individuals at a given timepoint. Sample size is the same between bins. Percent detection is defined as the percent of replicates where we found a significant difference between the old and young age bins. The red dashed line indicates when the bottleneck event occurred. The gray shaded portions of the plot indicate when samples were taken every 50 ticks of the simulation, where the unshaded portion of the plot indicates when samples were taking every five ticks of the simulation.

Supplemental Table 1: Mean sample sizes for annual and perennial simulations for age and temporal analyses. Values in parentheses represent the ranges in the data across replicates and timepoints. Annual sample sizes were calculated as the mean sample size for age sampling across perennial simulations.

|  |  | Bottleneck intensities | | |
| --- | --- | --- | --- | --- |
|  |  | 2 | 10 | 100 |
|  | 1 | 113 (111, 116) | 113 (111, 116) | 106 (103, 116) |
| Average age | 2 | 132 (128, 134) | 132 (129, 134) | 115 (109, 136) |
|  | 5 | 112 (110, 115) | 113 (110, 115) | 106 (103, 114) |
|  | 10 | 104 (99, 112) | 104 (100, 114) | 103 (96, 113) |
|  | 20 | 104 (98, 108) | 104 (99, 109) | 102 (98, 106) |
